# Phylogenetic predictions of carbapenemase activity from the Guiana extended-spectrum (GES) family of β-lactamases

**DOI:** 10.1093/jacamr/dlad150

**Published:** 2024-01-11

**Authors:** Miriam Barlow, Fred C Tenover

**Affiliations:** Department of Molecular and Cell Biology, University of California, Merced, CA 95343, USA; College of Arts and Sciences: Biology, University of Dayton, Dayton, OH 45469, USA

## Abstract

**Objectives:**

We investigated the amino acid substitutions in the GES family of ESBLs that were most likely to be involved in the evolution of carbapenemase activity.

**Methods:**

To identify the substitutions that are functionally important, we analysed the evolutionary history of the GES β-lactamases using an alignment and phylogeny to identify sites in GES that show evidence of positive selection and the selected phenotypes.

**Results and Conclusions:**

Data indicate that the substitutions G170S and G243A are associated with carbapenemase activity. The substitutions Q43E, E104K and T237A are most likely associated with ESBL activity.

## Introduction

The GES β-lactamase was first identified in a *Klebsiella pneumoniae* isolate collected in 1998.^1^ The biochemical characterization of that enzyme showed that it was an ESBL. Since that time, many variants of GES enzymes that differ in amino acid sequence have been isolated. While many GES variants continue to be implicated as ESBLs, some enzymes, such as GES-5, have been identified in carbapenem-resistant isolates^2^ and carbapenemase activity has been attributed to the GES enzyme. For some GES enzymes (e.g. GES 1–14, 19^3^), detailed biochemical studies have been conducted. However, for most GES enzymes, the studies implicating them as carbapenemases have mainly been descriptive, where clinical isolates are reported as resistant to carbapenems without any molecular or biochemical studies that specifically link the enzyme directly to the resistance phenotype. These results may be further confounded by differences in permeability and efflux systems in the organisms that contribute to carbapenem resistance. The finding that GES enzymes contribute to carbapenem resistance has also been confounded by the presence of other known carbapenemases, or carbapenemases genes, within the same isolates. Due to the presence of multiple resistance determinants and limited molecular biology studies, it is difficult to ascertain the phenotypic effects and functional changes that result from the amino acid substitutions observed in the various GES sequences that have emerged.

Other β-lactamases, such as TEM, SHV, CTX-M, KPC, OXA, AmpC and the MBLs NDM, VIM and IMP, have become significant threats to human health because of their widespread distribution in multiple bacterial species.^4^ Additionally, a more recently identified group, *bla*_GES_ genes are being reported with increasing frequency in several species,^5^ including *Klebsiella oxytoca, Pseudomonas aeruginosa*^2^ and *Acinetobacter* species. This is raising concern about the magnitude of the threat that GES β-lactamases may pose in the future as they demonstrate enhanced activity against carbapenems and disseminate among additional bacterial species. Given this, it is important to understand the functional significance of the novel mutations that are emerging and what contributions these enzymes are making to the microbial resistome in general and to carbapenem resistance specifically.

The objective of this study was to better understand which GES enzymes are ESBLs versus carbapenemases. We investigated which amino acid substitutions are functionally important for carbapenemase activity by assembling an evolutionary history of the GES β-lactamase family.

## Methods

### Sequence alignments

We downloaded the GES nucleic acid sequences from Genbank, translated them to amino acid sequences using the standard genetic code, and aligned them using the MUSCLE algorithm in MEGA with a gap-opening penalty of −2.90 and a gap-extension penalty of 0, and a hydrophobicity multiplier of 1.2. A complete list of the variant amino acids are shown in Table S1 (available as Supplementary data at *JAC-AMR* Online).

### Phylogenetic analysis

Using the MEGA software suite, we inferred a codon-based maximum-likelihood reconstruction of the phylogenetic history of the *bla*_GES_ gene variants using the default parameters (Tamura-Nei method with uniform rates across sites, the nearest-neighbour interchange maximum-likelihood heuristic search method, and no branch swap filter). Once the tree was reconstructed, we counted the numbers of unique lineages, along which each amino acid substitution has arisen, and catalogued those that have arisen more than once. We assumed that the multiple occurrences of those substitutions are best explained by positive selection. A list of GES β-lactamases, their variant amino acids at the sites under positive selection, number of independent occurrences of each substitution and our functional designations are shown in Table 1.

**Table 1.** Amino acid changes in GES β-lactamases and the resulting phenotypes

GES β-lactamases (accession)	Amino acid substitutions (number of independent occurrences)					Phenotypes	References
	Q43E (2)	E104K (5)	G170S (6)	T237A (2)	G243A (2)		
GES-1 AF156486						ESBL	^6^
GES-2 AF326355						Carbapenemase	^7^
GES-3 AB113580		X				Carbapenemase	^8^
GES-4 AB116723		X	X			Carbapenemase	^8^
GES-5 MF370188			X			Carbapenemase	^9^
GES-6 KM210290		X	X			Carbapenemase	^7^
GES-7 KX230795		X				ESBL	^7^
GES-8 AF329699						ESBL	^10^
GES-9 AY920928						ESBL	^11^
GES-10 FJ854362						Insufficient data	
GES-11 FJ854362						ESBL	^12^
GES-12 FN554543				X		ESBL	^12^
GES-13 GU169702		X				ESBL	^12^
GES-14 GU207844			X		X	Carbapenemase	^12^
GES-15 GU208678			X			Carbapenemase	^13^
GES-16 HM173356	X		X			Carbapenemase	^14^
GES-17 NG049119		X				Insufficient data	
GES-18 JQ028729			X			Carbapenemase	^15^
GES-19 JN596280					X	Carbapenemase	^16^
GES-20 JN596280			X			Carbapenemase	^16^
GES-21 JQ772478			X			Carbapenemase	^17^
GES-22 JX023441						Carbapenemase	^18^
GES-23 KF179354						Insufficient data	
GES-24 AB901141			X			Carbapenemase	^19^

						Inferred phenotype	
GES-25 KT732289			X			Carbapenemase
GES-27 KT151556			X			Carbapenemase
GES-28 KT626944			X			Carbapenemase
GES-29 KT997885			X			Carbapenemase
GES-30 LC126615			X			Carbapenemase
GES-31 KX034181	X	X				ESBL
GES-32 KX753225	X	X				ESBL
GES-33 KY783911		X	X	X		Carbapenemase
GES-34 MG748726		X	X			Carbapenemase
GES-36 LC385763			X			Carbapenemase
GES-37 LC385763			X			Carbapenemase
GES-38 MH780082		X				ESBL
GES-39 MH780083			X			Carbapenemase
GES-40 MK105833			X		X	Carbapenemase
GES-41 MK880227	X		X			Carbapenemase
GES-42 MN065796			X			Carbapenemase
GES-43 MN219691			X			Carbapenemase
GES-45 LC558356				X		ESBL
GES-46 MW295358		X				ESBL

## Assignment and inference of phenotype

To assign phenotypic effects to amino acid substitutions, we searched the available literature for each GES enzyme to identify both antimicrobial susceptibility testing data and biochemical (enzymatic) data to support the phenotypic assignments of enzymes as ESBLs or carbapenemases. We included a designation of ‘carbapenemase’ for enzymes reported to have carbapenemase activity.^9^ When no enzymatic activity was available, we used descriptions available for clinical isolates in which GES enzymes were identified. Carbapenemase activity in clinical isolates was described for isolates with carbapenem MICs that exceed clinical breakpoints.^20^ The specific information used to determine the activity of each GES β-lactamase can be found in the papers referenced in Table 1.

## Results

### Phylogenetic analysis

**Figure 1.**
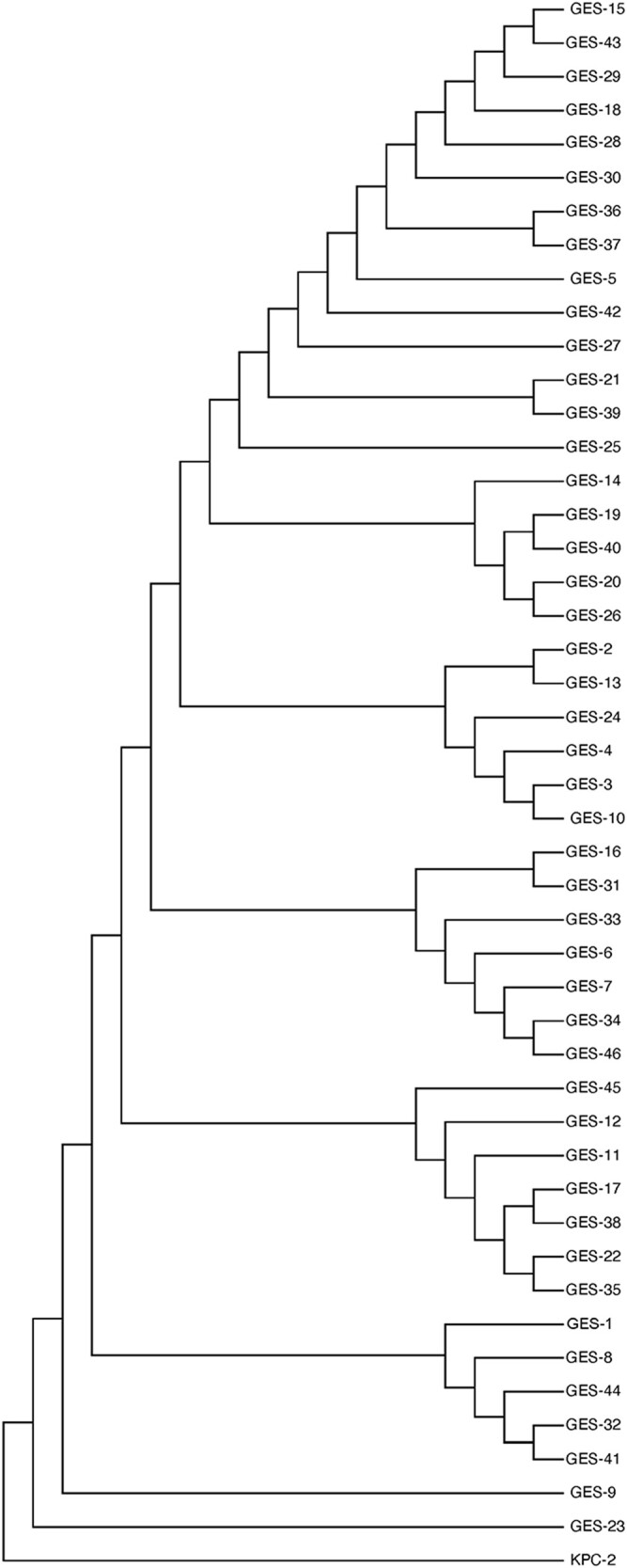
Phylogeny of *bla*_GES_ nucleotide sequences.

Phylogenetic analysis of the *bla*_GES_ nucleotide sequences confirmed that they are a monophyletic group that has been rapidly diversifying. A phylogenetic tree based on nucleotide sequences is shown in Figure 1. The *bla*_GES_ sequences have a mean similarity of 98%. We computed the overall dN/dS ratio as a test of positive selection and found that, on average, the *bla*_GES_ gene is consistently under purifying selection across all pairwise comparisons of alleles when all nucleotides are simultaneously considered. Most mutations that arise in the gene do not appear to be associated with carbapenemase activity and have no apparent functional effect in terms of enhancing spectrum of β-lactamase activity beyond that of an ESBL. However, when considered separately, there is the possibility that individual codons within the gene are still under positive selection. To detect such cases, we require a different test.

### Analysis of amino acid substitutions

To identify possible singular incidences of positive selection, amino acid substitutions that have been selected independently more than once in the evolutionary history of GES β-lactamases were identified as previously described.^21^ Briefly, when an evolved trait, including a mutation, arises independently multiple times there is generally strong selection from the environment for evolution of that trait, resulting in positive selection. By using a phylogenetic reconstruction of the evolutionary history of the gene or species being considered, it is possible to enumerate how many times each trait has arisen and to identify those that are responsive to strong environmental selective pressures.

There were five amino acid substitutions that showed evidence of positive selection: G170S, G243A, Q43E, E104K and G243A (Table 1). Using these five substitutions, we were then able to assess their reliability as molecular indicators of carbapenemase activity using reported phenotypes of other GES enzymes (Table 1). We identified phenotypes reported in previous studies (Table 1) to categorize the GES enzymes into ESBL and carbapenemase phenotypes. Among published reports, isolates expressing GES-5 consistently exhibit higher carbapenemase activity than results reported from expression of other GES enzymes. GES-5 contains the substitution G170S, which is associated with carbapenemase activity. The G243A substitution is also associated with carbapenemase activity, but reported K_cat_/k_m_ activity for GES enzymes containing the G243A substitution is 90% lower than GES-5^9^ and MICs of carbapenems are lower when compared with GES-5 (Table 1). For E104K, there is some ambiguity about its role in carbapenemase activity because GES-3 and GES-13 both contain this substitution. While GES-3 exhibits carbapenemase activity, GES-13 does not. Both of these enzymes contain other substitutions that are clearly modulating any influence held by E104K. Substitutions Q43E and T237S also have similar ambiguity, where they on their own do not appear to confer carbapenemase activity, but they are found in combinations with other substitutions in enzymes exhibiting carbapenemase activity. The substitutions Q43E, E104K and T237S are not good predictors of carbapenemase and they may or may not interact with other substitutions to contribute to carbapenem resistance. Future experimental studies are necessary to test the accuracy of these predictions.

## Discussion

As with most antimicrobial resistance genes, the evolution of sequences encoding the GES β-lactamases resulting in carbapenemase activity is directed by positive selection. When adaptation is occurring in response to a selective pressure, evolutionary convergence occurs, meaning that the advantageous adaptations, which in this case are amino acid substitutions resulting in elevated carbapenemase activity, will independently arise multiple times. GES β-lactamases comprise a monophyletic group of class A serine β-lactamases that are located either in the chromosomes or on plasmids of many species including *Klebsiella* species, *Pseudomonas* species and *Acinetobacter* species.^9^ Due to their single origin, they share nearly identical sequences, which differ by very few amino acids. The seemingly recent divergence of these enzymes makes it likely that the amino acid sequences are not saturated with substitutions, and that the inference of their evolutionary history will be straightforward. The low number of substitutions that differ between enzymes enable us to determine how many times each substitution has occurred throughout the evolution of the GES enzymes. By inferring which substitutions have occurred multiple times in isolates that are carbapenem resistant, we can obtain a list of candidate substitutions that are involved in carbapenem resistance. For confirmation of the functional effect, the substitutions should be introduced into cloned *bla*_GES_ genes and their phenotypic and biochemical affects assessed. We have not conducted those studies.

The G170S substitution has been previously characterized as resulting in detectable imipenem hydrolysis when combined with the E104K substitution in GES-4.^22^ The G170S substitution likewise results in elevated resistance to imipenem and meropenem when it is the only substitution that differs from GES-1, as is seen in GES-5,^9^ confirming its role in carbapenemase activity. Whether the G243A mutation, which is associated with carbapenemase activity, is clinically relevant and would lead to treatment failure is unknown. Pharmacokinetic studies in animal studies may provide some insights into this issue.

A limitation of this study is that we did not conduct laboratory experiments to confirm the functional effect of each substitution by introducing them into cloned *bla*_GES_ genes and elucidating their phenotypic and biochemical effects. Greater experimental characterization of these substitutions will be a worthwhile future direction.

## Supplementary Material

dlad150_Supplementary_Data

## Supplementary data

Table S1 is available as Supplementary data at *JAC-AMR* Online.
